# Whey Protein–Quercetin–Gellan Gum Complexes Prepared Using pH-Shift Treatment: Structural and Functional Properties

**DOI:** 10.3390/foods14152720

**Published:** 2025-08-03

**Authors:** Na Guo, Xin Zhou, Ganghua Zhou, Yimeng Zhang, Guoqing Yu, Yangliu Liu, Beibei Li, Fangyan Zhang, Guilan Zhu

**Affiliations:** 1 Department of Biological and Food Engineering, Hefei Normal University, Hefei 230061, China; 2 Anhui Green Food Rural Revitalization Collaborative Technology Service Center, Hefei 230061, China

**Keywords:** quercetin, whey protein, pH-shift

## Abstract

The objectives of this study were to prepare whey protein–quercetin–gellan gum conjugates using the pH-shift method and to evaluate the impacts of varying pH values and quercetin concentrations on the interaction mechanisms and functional characteristics of the complexes. Spectroscopic analyses (fluorescence, UV-vis, and FT-IR) revealed that new complexes formed under alkaline conditions. Notably, an increasing quercetin concentration led to a reduction in complex particle size and an increase in the zeta potential value, with these effects being more pronounced under alkaline conditions. The particle size was 425.7 nm, and the zeta potential value was −30.00 mV at a quercetin addition concentration of 15 umol/g protein. Additionally, the complexes formed under alkaline conditions exhibited superior foaming capacity, emulsification properties, and significantly enhanced free radical scavenging activity. The complex’s DPPH and ABTS radical scavenging rates rose by 41.57% and 57.69%, respectively. This study provides theoretical foundations and practical insights for developing protein—polyphenol systems, offering significant implications for the application of quercetin functional foods and supplements in the food science and pharmaceutical industries.

## 1. Introduction

Quercetin is a natural flavonoid widely distributed in dietary foods such as onions, tomatoes, and apples. Owing to its potential health benefits, such as antioxidant, antitumor, hypoglycemic, and anti-obesity activities, it has gained substantial attention in the development of functional nutritional supplements [1]. However, it exhibits extremely poor water solubility, which leads to its difficult absorption and utilization by the human body, thereby restricting its application in the field of functional foods. Quercetin molecules have several hydrophilic hydroxyl groups, which enable them to form non-covalent or covalent interactions with polysaccharides and proteins in the food matrix [2]. Therefore, many researchers have explored the formation of nanocomplexes between quercetin and biopolymers as a strategy to improve its bioavailability. For instance, the encapsulation of hydrophobic quercetin in zein protein/soluble soybean polysaccharide nanoparticles has been shown to significantly enhance its photochemical stability and ABTS radical scavenging activity [3]. Carboxymethyl chitosan/tannic acid hydrogels integrated with quercetin-loaded zeolitic imidazolate frameworks enable the localized delivery of quercetin for diabetic wound healing [4].

Whey proteins have excellent water solubility and emulsifying, foaming, and gelling properties [5], and they have natural hydrophobic pockets in their structure that can serve as binding sites for lipid-soluble molecules [6]. Therefore, whey proteins are widely used to construct polyphenol delivery vehicles. For example, the interaction between whey isolate proteins and anthocyanins enhances the latter’s color stability and antioxidant activity [7]. The binding of whey protein concentrate to quercetin has been shown to enhance the latter’s DPPH radical scavenging and reducing abilities [8]. Whey proteins can bind to polyphenols in both reversible and irreversible ways. Reversible binding is mainly driven by self-assembly interactions, such as hydrogen, hydrophobic, and ionic bonding, whereas irreversible interactions are mainly formed using alkali-driven [9], chemical coupling, and free radical-mediated methods [10]. Currently, the pH-shift treatment of the protein–polyphenol co-assembly technique, which has the advantages of simplicity, environmental friendliness, and low cost, is attracting considerable attention from researchers. In particular, pH modulation alters the structure of proteins, leading to the unfolding of their spatial structure and enhancement of the solubility, surface hydrophobicity, emulsification, and antioxidant properties of the complexes, as well as the binding capacity of the proteins to the polyphenols (EGCG, rose anthocyanin) [11,12].

Due to the poor tolerance of proteins and polyphenols to the gastric environment, whey protein–quercetin complexes have been prepared and encapsulated with gellan gum to enhance their strength and stability [13]. At present, there is no detailed theoretical analysis of the interaction between quercetin and whey protein or polysaccharides regulated by different pH values. Therefore, the aims of the present study were as follows: to prepare whey protein–quercetin–gellan gum complexes with different interactions and quercetin concentrations using a simple and green pH-induced protein–polyphenol binding technique; to investigate the basic physicochemical and functional properties of the complexes; and to provide a theoretical basis for the development and application of ternary complexes of proteins, polyphenols, and polysaccharides.

## 2. Materials and Methods

### 2.1. Materials and Reagents

Whey protein (WPI, Hilmar™ 9410) was purchased from Hilmar Cheese Company (Hilma, CA, USA). Quercetin (Q) was acquired from Boomerang Biological Company (Hefei, Anhui, China). Gellan gum (GG, pH = 6.7 and 96.5% purity with a gel strength of 655 g/cm^2^) was obtained from Bomei Company Co., Ltd. (Hefei, Anhui, China). All other reagents used in this study were analytically pure.

### 2.2. Experimental Methods

#### 2.2.1. Preparation of Ternary Complexes

##### Preparation of Whey Protein–Quercetin Complexes

A 2% (m/V) whey protein solution (dissolved in water) and a 5 mg/mL quercetin solution (dissolved in 75% ethanol) were prepared. The pH of the protein solutions was adjusted to 7.4 and 9.0 with 0.1 M NaOH. The whey protein and quercetin solutions were mixed to obtain three solutions with quercetin concentrations of 0, 5, and 15 μmol/g protein according to our preliminary research (S1). The mixtures were continuously stirred for 24 h at room temperature in open air. The samples were dialyzed for 48 h using dialysis bags (MWCO 8000–14,000 Da) to remove free quercetin and freeze-dried to obtain whey protein–quercetin complexes.

##### Preparation of Whey Protein–Quercetin–Gellan Gum Complexes

The whey protein–quercetin complex was dissolved in deionized water to a concentration of 20 mg/mL and stirred at 25 °C overnight. The solution was thoroughly mixed with gellan gum at 25 °C while ensuring that it contained 0.1% (m/V) gellan gum. The samples were dialyzed for 48 h and freeze-dried to obtain a ternary complex of whey protein–quercetin–gellan gum. These complexes were named pH7.4-Q_0_, pH7.4-Q_5_, pH7.4-Q_15_, pH9.0-Q_0_, pH9.0-Q_5_, and pH9.0-Q_15_ (the subscript numbers represent the quercetin concentrations).

#### 2.2.2. SDS-PAGE Analysis

SDS-PAGE electrophoresis was performed using a 5–20% polyacrylamide gel. The concentration of the concentration gel was 5%, and that of the separation gel was 20%. The diluted sample solution (1 mg/mL) was mixed with the uploading buffer, and all samples were heated in a boiling water bath for 5 min. Then, 10 μL of the samples was placed in each well, and protein markers (11–180 kD) were used (Soleberg, Beijing, China). The samples were stained and fixed using R-250 solution and destained.

#### 2.2.3. Endogenous Fluorescence Spectroscopy Analysis

A 0.2 mg/mL protein sample was prepared with deionized water, and changes in the tryptophan residues in the samples were measured in the spectral range of 300–450 nm using a fluorescence spectrophotometer (Shimadzu RF5300, Shimadzu, Japan). The excitation and emission slit widths were kept at 10 nm during the scanning process. The excitation wavelength was 280 nm, with a scanning speed of 240 nm/min and a detection resolution of 1 nm.

#### 2.2.4. Ultraviolet-Visible (UV-Vis) Absorption Spectroscopy Analysis

The UV-vis absorption spectra (UV1800, Shimadzu, Japan) of different sample solutions with a protein concentration of 1 mg/mL were recorded in the wavelength range of 200–450 nm with a detection resolution of 0.2 nm. Deionized water was used as a blank control.

#### 2.2.5. Fourier Transform Infrared Spectra

The lyophilized samples were scanned in the infrared band (4000–500 cm^−1^) using the ATR mode of a Fourier transform infrared spectrometer (IS20, Thermo Scientific Nicolet, Madison, WI, USA) for 32 scans, with a resolution of 4 cm^−1^.

#### 2.2.6. Measurement of Particle Size and Potential

The particle size and potential of the complexes were measured using a laser particle sizer (90Plus PALS, Brookhaven, New York, NY, USA). The temperature was 25 °C, and the refractive indices of the protein particles and dispersion medium were 1.46 and 1.33, respectively. Each sample was analyzed thrice, and the results are expressed as mean values.

#### 2.2.7. Determination of Polyphenol Binding Capacity

A standard calibration solution of gallic acid was prepared. The complexes were dialyzed at 4 °C for 12 h. Then, 4 mL of the solution outside each dialysate was mixed with 4 mL of distilled water and 0.5 mL of forintol reagent, and the mixture was left to stand for 5 min. Next, 1.5 mL of 20% sodium carbonate solution was added and shaken well. The mixture was incubated for 2 h in the dark at room temperature. Then, the absorbance was measured at 765 nm using a spectrophotometer, with distilled water used as a blank. The formula for the gallic acid standard curve is y = 0.0022x + 0.0013, with R^2^ = 0.9993. The formula for calculating the proportion of bound quercetin is shown in Equation (1):

(1) $$Bound\;quercetin\;(\%)=\frac{total\;quercetin\;quality-quercetin\;content\;of\;extradialytic\;fluid}{\;total\;quercetin\;quality}\times 100\%$$

#### 2.2.8. Determination of Turbidity of the Complexes

The absorbance value of each sample was determined at 600 nm by taking the freeze-dried solution of the complex and using deionized water as a blank control. For each sample, the analysis was repeated thrice, and the average of the measured absorbance values was calculated.

#### 2.2.9. Determination of the Emulsifying Ability of the Complexes

##### Foaming

Briefly, 3 mL of each sample was homogenized using an emulsifying homogenizer (A30, Ouhor, Shanghai, China) for 1 min at 11,000 rpm. The volumes at 0 min and 10 min were determined, and the foaming capacity (FC) and foaming stability (FS) were calculated using Equations (2) and (3):

(2) $$FC\;(\%)=\frac{\;V_{0}-3}{\;3}\times 100\%$$

(3) $$FS\;(\%)=\frac{\;V_{t}-3}{V_{0}-3}\times 100\%$$

where *V*_0_ is the volume at 0 min after whipping, and *V_t_* is the volume at 10 min after whipping.

##### Emulsification

Briefly, 3 mL of each sample was mixed with 1 mL of soybean oil and homogenized for 1 min at 11,000 rpm. Next, 40 μL of the bottom solution was taken at 0 min and 10 min and mixed with 5 mL of 0.1% sodium dodecyl sulfate solution. The absorbance of the resultant solution was determined at 500 nm, and the volumes were calculated to determine the emulsion stability index (ESI) and emulsifying activity index (EAI) using Equations (4) and (5):

(4) $$EAI\;(m^{2}/g)=\frac{2\;\times \;2.303\;\times \;A_{0}\;\times \;DF}{\;1000\;\times \;Ø\;\times \;L\;\times \;C\;}$$

(5) $$ESI\;(min)=\frac{\;A_{0}\times 10}{\;A_{0}-A_{10}}$$

where *DF* is the dilution factor; *C* is the concentration of protein in the sample (g/mL); *L* is the optical path length of the cuvette (1 cm); Ø is the proportion of the oil phase to the sample (0.25); and *A*_0_ and *A*_10_ are the absorbances of the sample at 0 min and 10 min.

#### 2.2.10. Thermal Analysis of Complexes

The temperature at which the mass of each substance in the sample was lost was detected using a simultaneous thermal analyzer (SDT650, TA Instruments, New Castle, DE, USA). Briefly, 6 mg of the sample was collected in an alumina crucible and subjected to a temperature program of 35–500 °C, with a temperature increase rate of 10 °C/min. The experiment was carried out under a nitrogen flow rate of 100 mL/min to prevent the sample from reacting with air, which could affect the substance’s structure.

#### 2.2.11. Scanning Electron MICROSCOPY (SEM) of Complexes

The microstructure of the sample was observed using a scanning electron microscope (EVO MA-15, ZEISS, Oberkochen, Baden-Württemberg, Germany) at a magnification of 500×.

#### 2.2.12. Determination of the Antioxidant Properties of Compounds

##### DPPH Radical Scavenging Rate

Briefly, a 1.75 × 10^−4^ mol/L DPPH solution was prepared using ethanol. Then, 2 mL of each sample solution (0.5 mg/mL) was mixed with 2 mL of DPPH ethanol solution, and the reaction mixture was incubated for 1 h at room temperature in the dark. Then, the absorbance of the solution was detected at 517 nm. Equation (6) was used to calculate the DPPH radical scavenging rate of the sample:

(6) $$\mathrm{Free}\;\mathrm{radical}\;\mathrm{scavenging}\;\mathrm{rate}\;\left(\%\right)\;=\;\frac{A_{c}\;-\;A_{s}}{A_{c}}\;\times \;100\%$$

where *A_c_* is the absorbance value of the solution containing the sample (after the reaction), and *A_s_* is the absorbance value of the solution without the sample.

##### ABTS Radical Scavenging Rate

Briefly, a 7 mmol/L aqueous ABTS solution was mixed with a 2.45 mmol/L potassium persulfate solution, and the mixture was incubated for more than 12 h in the dark. Before the experiment, the ABTS reserve solution was diluted with methanol until its absorbance at 747 nm reached 0.7 ± 0.02. Next, 1 mL of the extract diluted to a certain concentration was added to the ABTS solution in a 1:3 (*v*/*v*) ratio. The reaction mixture was shaken for 20 s and then reacted for 60 min. The absorbance of the resultant solution was measured at 734 nm, and the ABTS radical scavenging rate of the sample was calculated using Equation (6).

### 2.3. Statistical Analysis

Data were analyzed using the SPSS 26 statistical software (IBM, Armonk, NY, USA) and an ANOVA test with a significance level of *p* < 0.05. Origin 2021 (OriginLab, Northampton, MA, USA) was used to analyze and graph the experimental data. Each experiment was performed in triplicate.

## 3. Results and Discussion

### 3.1. SDS-PAGE

**Figure 1:**
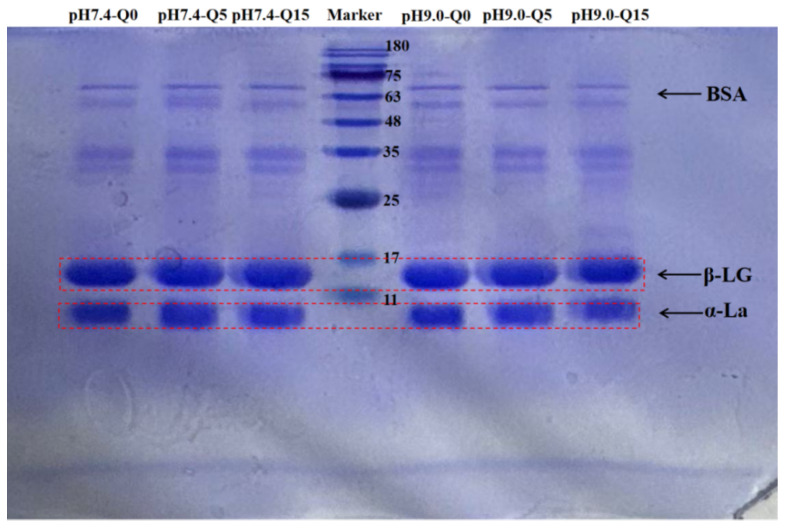
SDS-PAGE of the complex.

Variations in the binding of whey proteins to quercetin at different pH values were investigated using SDS-PAGE. As shown in Figure 1, there was no significant change in the subunit bands of whey proteins at pH 7.4, which was attributed to their reversible bonding to quercetin under these conditions. However, at pH 9.0 and a quercetin concentration of 15 μmol/g protein, the subunit bands of β-LG and α-La slightly shifted upward, suggesting that quercetin covalently bound to both the β-LG and α-La proteins of whey [14].

### 3.2. Analysis of Endogenous Fluorescence Spectra

**Figure 2:**
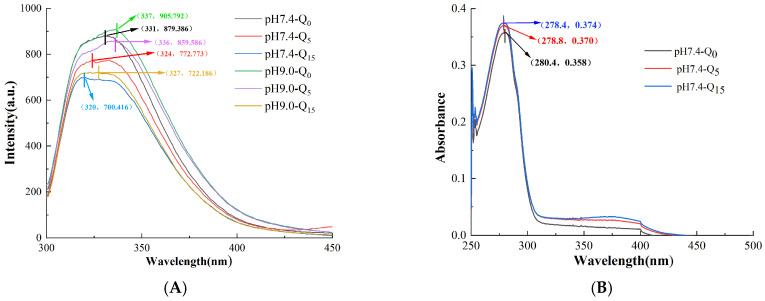

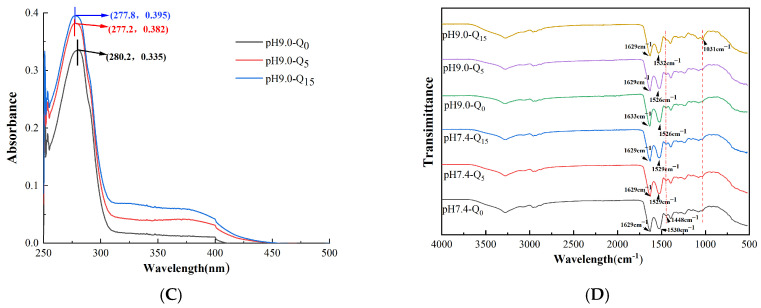
Fluorescence spectrum (**A**), UV-vis absorption spectra of complex solutions at pH 7.4 (**B**) and pH 9.0 (**C**), and infrared spectral analysis (**D**) of the complex.

Figure 2A shows fluorescence spectra of the complexes prepared at different pH values and with varying quercetin concentrations. The tryptophan and tyrosine residues in whey proteins were found to emit fluorescence of a certain intensity at specific excitation wavelengths. The binding of the complexes was indicated by comparing the fluorescence intensities and wavelength migration corresponding to the peaks of the maximum fluorescence intensity [15]. We observed that the fluorescence intensity of the complexes decreased with an increasing quercetin concentration, indicating that quercetin binding led to fluorescence quenching of the protein [16]. Moreover, with an increasing quercetin concentration, the λ_max_ of the complexes synthesized under neutral conditions clearly blue-shifted by 7 nm and 11 nm, and the complexes synthesized under alkaline conditions blue-shifted by 1 nm and 16 nm, respectively. This result indicates that quercetin, whey proteins, and gellan gum interact with each other. Moreover, the interactions between quercetin and tryptophan were stronger under alkaline conditions [9].

### 3.3. UV-Vis Absorption Spectra

Figure 2B,C shows UV-vis absorption spectra of the complexes prepared at different pH values and with varying quercetin concentrations. Whey protein exhibited a distinct absorption peak at 280 nm, which might be attributed to the aromatic amino acid residues in the protein, such as tryptophan and tyrosine, which contain conjugated double bonds [17]. After the introduction of quercetin and gellan gum into the complex, the absorbance gradually enhanced with an increasing quercetin concentration. This finding indicates a change in the microenvironment of the system, where the addition of quercetin exposed the aromatic heterocyclic hydrophobic groups in the aromatic residues of the protein, such as tryptophan and tyrosine, leading to enhanced absorbance of the complex [1]. We observed that the complexes blue-shifted by 1 nm and 1.4 nm under neutral conditions and by 2.8 nm and 2.6 nm under alkaline conditions, suggesting potential interactions among quercetin, whey protein, and gellan gum [7]. In addition, at the same quercetin concentration, the intensity of the UV absorption peak of the complex was significantly higher at pH 9 than at pH 7.4, which might be attributed to the stronger binding under alkaline conditions [17].

### 3.4. Infrared Spectral Analysis

As shown in Figure 2D, the absorption bands of the complex at 1629 cm^−1^ and 1526 cm^−1^ represent the protein C = O stretching vibration (amide absorption band Ⅰ) and N-H stretching vibration (amide absorption band Ⅱ) [18].

Infrared spectral changes in the three complexes at pH 7.4 were not noticeable. However, at pH 9.0, the amide Ⅰ band shifted from 1629 cm^−1^ to 1633 cm^−1^, indicating that the alkaline environment affected the intermolecular forces. Moreover, after adsorbing quercetin, the peak wavenumber of the protein amide II band of the complex gradually shifted from 1526 cm^−1^ to 1532 cm^−1^, while the wavenumber of the amide I band shifted from 1633 cm^−1^ to 1629 cm^−1^. This phenomenon might result from hydrogen bonding between quercetin and the protein [19].

In particular, a new transmission peak appeared at a wavenumber of 1031 cm^−1^ for the complex pH9.0-Q_15_. This may be because, under alkaline conditions, the quinone compounds formed by the oxidation of quercetin are more likely to form irreversible C-N bond stretching vibrations with the thiol or amino groups in proteins. In addition, the shoulder peak disappeared at 1448 cm^−1^, indicating that a new covalent bond interaction formed between quercetin and the protein.

### 3.5. Particle Size and Zeta Potential

**Figure 3:**
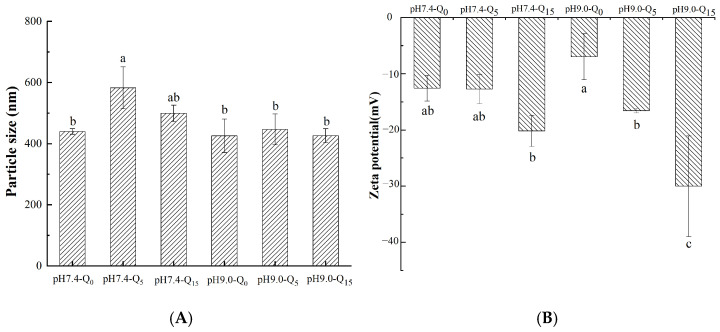

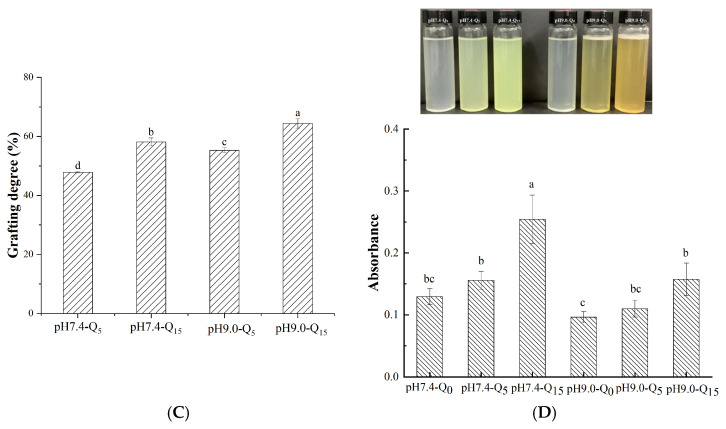
Particle size (**A**), zeta potential (**B**), polyphenol grafting degree (**C**), and turbidity (**D**) of the complexes. Different lowercase letters indicate significant differences between groups (*p* < 0.05).

The particle size and zeta potential of the complexes prepared at different pH values and with varying quercetin concentrations are shown in Figure 3A,B. We observed that the addition of quercetin changed the particle size of the ternary complexes, indicating that the interactions among whey protein, quercetin, and gellan gum influenced the particle size of the complexes. The particle size of the ternary complex showed a decreasing trend with an increasing quercetin concentration, indicating the formation of a stable complex. This decrease in particle size might be attributed to the molecular interactions between quercetin and the protein, resulting in a more compact protein structure [9]. Furthermore, the particle size of the ternary complexes was significantly lower under alkaline conditions than under neutral conditions. This finding might be attributed to the irreversible interactions between whey protein and quercetin under alkaline conditions, making whey protein, quercetin, and gellan gum more tightly linked than during reversible interactions [20]. Figure 3B shows that the zeta potentials of the whey protein–gellan gum binary complexes were −12.57 mV and −6.95 mV at pH 7.4 and pH 9, respectively. After the addition of quercetin, the absolute values of the zeta potentials increased with an increasing quercetin concentration, varying from −12.57 mV to −20.17 mV at pH 7.4 and from −6.95 mV to −30 mV at pH 9. The larger complex zeta potential was associated with quercetin-induced structural changes in the protein, leading to the exposure of some polar groups [21]. Moreover, the absolute zeta potential values of the ternary complexes under alkaline conditions were higher than those under neutral conditions; this may be related to the partial oxidation of quercetin at pH 9.0, as negatively charged quinones formed, and the complex system’s negative charge increased with an increasing quercetin concentration. This finding indicates that the stability of irreversible bound complex solutions was better than that of reversible bound complex solutions. This finding is in agreement with the particle size assessment results.

### 3.6. Polyphenol Binding Capacity

The polyphenol binding capacity can reflect the affinity of the irreversible and reversible interactions of quercetin. As shown in Figure 3C, the quercetin binding capacity of the complex increased with an increasing quercetin concentration, with a significantly higher capacity under alkaline conditions than under neutral conditions. These results indicate that the irreversible force was weaker than the reversible force in terms of quercetin binding. This finding might be attributed to the oxidation of catechol in the quercetin structure to quinone under alkaline conditions, which interacts with whey protein and gellan gum to make the ternary complex stronger. These phenomena led to the differences in the polyphenol binding capacities of the complexes under the two pH conditions [22].

### 3.7. Turbidity

Turbidity refers to the degree of light obstruction by suspended material, reflecting the aggregation of proteins in the system. In addition, the turbidity of a solution can be directly reflected in its appearance. As shown in Figure 3D, with the same quercetin concentration, the complex solution at pH 9 showed a clearer and more transparent state with a significantly lower turbidity than that at pH 7.4. This result might be attributed to the irreversible interactions within the complex at pH 9, which enable the binding of more quercetin in the system, resulting in a lower amount of free quercetin. It might also be attributed to the better solubility of proteins under alkaline conditions. In addition, the formation of ternary complexes increases the weight of particles, enhancing the solution’s turbidity [23].

### 3.8. Emulsification Properties

**Figure 4:**
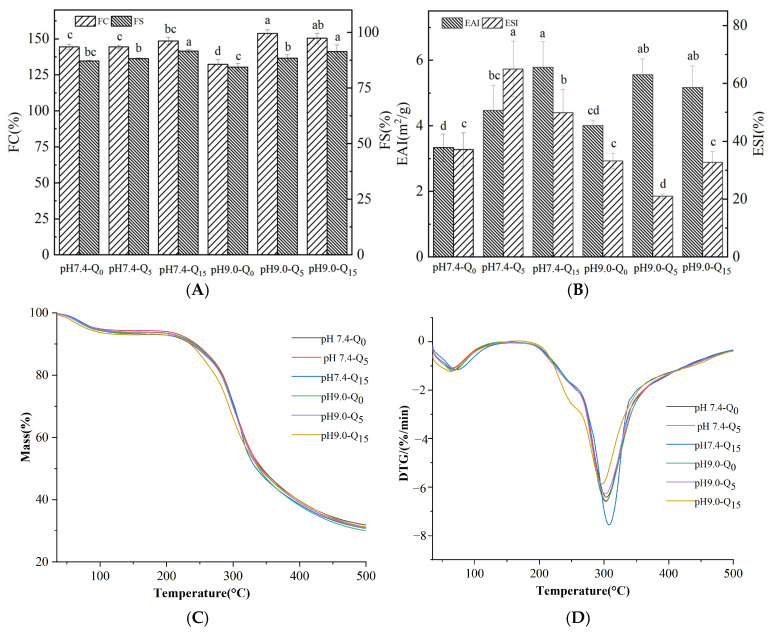
Foaming properties (**A**), emulsification properties (**B**), TGA thermograms (**C**), and DTG thermograms (**D**) of the complexes. Different lowercase letters indicate significant differences between groups (*p* < 0.05).

The FC (foaming capacity) and FS (foaming stability) of the complexes at different pH values and with varying quercetin concentrations are shown in Figure 4A. The whey protein–gellan gum complex exhibited good foaming properties, and the relatively weak reversible force between whey protein and quercetin only mildly impacted the FC. However, the FC values of the irreversible complexes significantly increased with an increasing quercetin concentration (*p* < 0.05). The enhancement of the foaming properties of both complexes was associated with changes in the secondary structure of the proteins, which enhanced the affinity of the proteins for the oil/water interface, which, in turn, affected the dispersion efficiency of the proteins at the air/water interface [24]. Furthermore, the irreversible complexes exhibited a significantly higher foaming ability than the reversible complexes (*p* < 0.05). This result might be attributed to the greater impact of the irreversible interactions between whey proteins and quercetin on the protein structure. The significant increase in FS values with both quercetin concentrations might be attributed to the surface potential of the proteins. The zeta potentials of the two complexes increased with an increasing quercetin concentration, with higher surface zeta potentials improving the stability of the protein membrane and forming a stable interfacial membrane with enhanced elasticity at the gas/liquid interface [25].

The EAI and ESI of the complexes with different pH and quercetin concentrations are shown in Figure 4B. Our results show that the quercetin-containing complexes exhibited significantly higher EAI values than the complexes lacking quercetin (*p* < 0.05). Both reversible and irreversible binding with quercetin altered the protein secondary structure, thereby increasing the molecular flexibility of the protein [12]. In addition, the addition of quercetin to the complexes decreased the stability of the irreversible complexes but increased that of the reversible complexes. The decrease in the ESI of the reversible complexes might be attributed to the increase in their spatial rigidity, which reduced the flexibility of the protein and, hence, the emulsification properties of the complexes [10,26].

### 3.9. Thermal Analysis

As shown in Figure 4C, the TGA curves of the reversible and irreversible complexes were similar, with two significant weight loss events in the temperature range of 35–400 °C. The weight loss event within the 35–100 °C range corresponded to the evaporation of free and bound water [27]. The weight loss event within the 200–400 °C range corresponded to the degradation of the protein structure and the thermal degradation of phenolics and polysaccharide chains [28]. The maximum thermal decomposition rate (Tmax) of the reversible complexes is shown in Figure 4D, and it was similar to that of the irreversible complexes. The results revealed no significant difference between the effects of irreversible and reversible binding on the melting peak temperature of the whey protein–quercetin–gellan gum complexes [12].

### 3.10. Scanning Electron Microscopy

**Figure 5:**
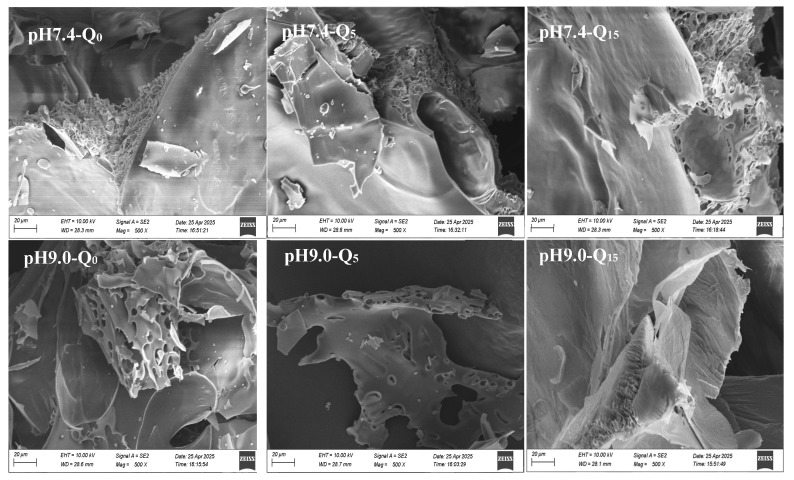
Scanning electron micrographs of the complexes at 500×, respectively.

Figure 5 shows SEM images of the complexes at different pH and quercetin concentrations. The cross-sections of the ternary complexes showed a tightly stacked structure. With an increasing quercetin concentration, the steric void structure of the reversible complexes progressively loosened, while that of the irreversible complexes became denser. The pores of the reversible complex became larger and looser, indicating that the increasing quercetin concentration led to the enhancement of electrostatic repulsion between the complex molecules [29]. However, the structure of the irreversible complexes became more compact and the pores became smaller with the increasing quercetin concentration, indicating a higher cross-linking density between the complex molecules. This finding suggests a strong interaction force between the whey protein–quercetin complex (formed by irreversible binding) and gellan gum [30].

### 3.11. Antioxidant Properties

**Figure 6:**
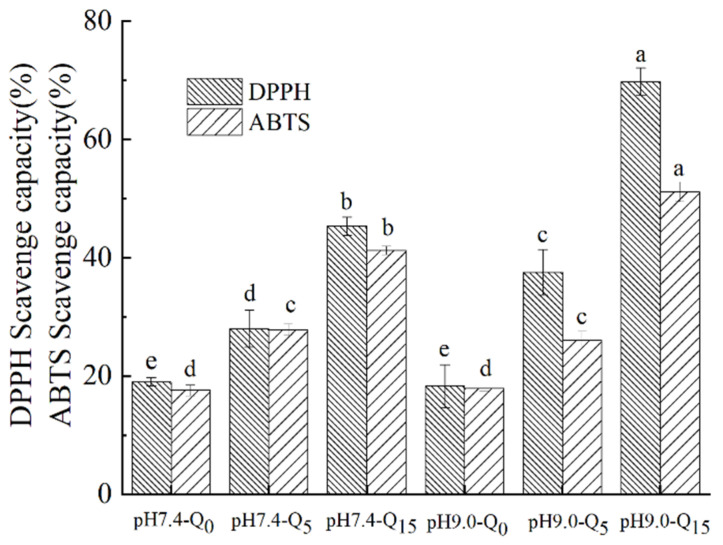
DPPH and ABTS scavenging abilities of the complexes. Different lowercase letters indicate significant differences between groups (*p* < 0.05).

As can be seen in Figure 6, the whey protein–gellan gum binary complexes possessed free radical scavenging abilities. However, the DPPH and ABTS scavenging rates of the ternary complexes containing quercetin (15 μmol/g protein) increased by 57.69% and 41.57% at pH 9 and by 26.33% and 29.56% at pH 7.4, respectively, compared to those of the whey protein–gellan gum binary complex. Furthermore, the DPPH and ABTS radical scavenging capacities of the complexes increased with an increasing quercetin concentration. The addition of quercetin increased the proton and electron donor levels in the complex, significantly improving the antioxidant activity of the complex [31]. In addition, the complex solutions at pH 9 exhibited higher free radical scavenging rates than those at pH 7.4, suggesting that the complexes prepared via the irreversible binding of the three components at pH 9 exhibited better antioxidant activities [10].

## 4. Conclusions

Polyphenols, proteins, and polysaccharides are important components of the human diet, and they coexist in food production and processing. It is crucial to study the stability of these three components in complex food systems and their impact on product quality. In this study, a whey protein–quercetin–gellan gum ternary complex prepared using the pH-shift method easily formed irreversible binding under alkaline conditions, and its structural stability and antioxidant capacity significantly improved. As a safe dietary supplement, quercetin has broad application prospects, but extensive research is still needed to examine its optimal application methods, especially in functional foods made by embedding it with biological macromolecules, and the safety of its long-term intake.

## Data Availability

The original contributions presented in the study are included in the article/Supplementary Material, further inquiries can be directed to the corresponding authors.

## Supplementary Materials

The following supporting information can be downloaded at: https://www.mdpi.com/article/10.3390/foods14152720/s1, Figure S1:Emulsion stability of the complexs of binary complexes (Whey protein-Quercetin) and ternary complexes.
