# Supplementary material for: Whey Protein–Quercetin–Gellan Gum Complexes Prepared Using pH-Shift Treatment: Structural and Functional Properties
Source: Foods. 2025 Aug 3;14(15):2720. doi: 10.3390/foods14152720 (PMC12346554; doi:10.3390/foods14152720)
Supplement: Supplementary file 1 [file foods-14-02720-s001.zip › foods-3779457-supplementary.pdf]

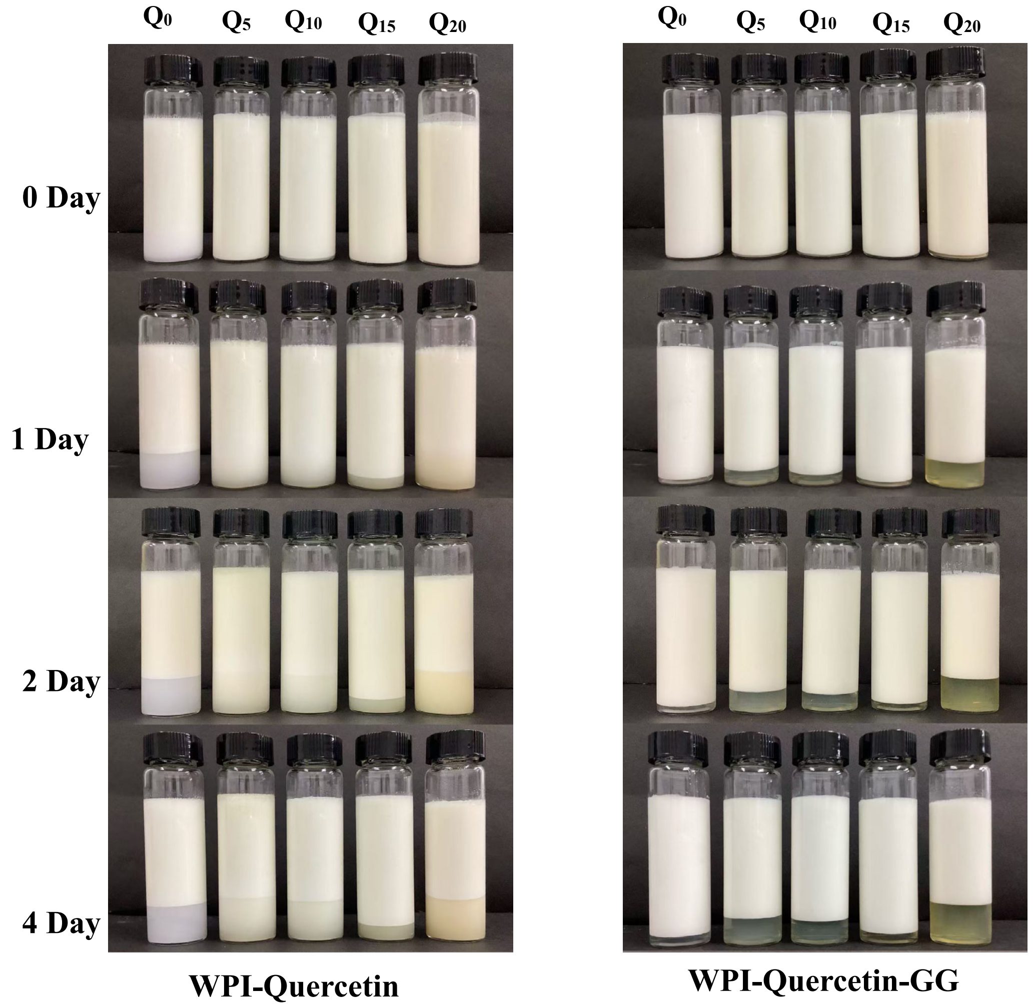

Figure S1. Emulsion stability of the complexs of binary complexes (Whey protein-Quercetin) and ternary complexes (Whey protein-Quercetin-gellan gum) (Q_0, Q_5, Q_{10}, $Q_{15,}$ Q_{20} represent quercetin concentrations of 0, 5, 10, 15, 20 μmol/g protein)
